# Microanatomic Morphometric Characteristics of the Third Ventricle Floor

**DOI:** 10.3390/brainsci13040580

**Published:** 2023-03-29

**Authors:** Marios Theologou, Konstantinos Kouskouras, Konstantinos Natsis, Panagiotis Varoutis, Eleni Zaggelidou, Christos Tsonidis

**Affiliations:** 1 Second Department of Neurosurgery, General Hospital of Thessaloniki Hippokratio, Aristotle University of Thessaloniki, 54642 Thessaloniki, Greece; 2 Department of Radiology, AHEPA University Hospital, Aristotle University of Thessaloniki, 54636 Thessaloniki, Greece; 3 Department of Anatomy and Surgical Anatomy, School of Medicine, Aristotle University of Thessaloniki, 54124 Thessaloniki, Greece; 4 Forensic Medical Service of Thessaloniki, Ministry of Justice, 54634 Thessaloniki, Greece

**Keywords:** endoscopic third ventriculostomy, mamillary body, hypothalamus, basilar artery, Liliequist membrane, lamina terminalis

## Abstract

**Background**: Endoscopic third ventriculostomy (ETV) is an effective treatment for hydrocephalus. The in-depth understanding of microanatomy is essential for accurate diagnosis, treatment and complications prevention. The aim of this study is to supplement the knowledge gap regarding the microanatomical metrics and correlations for which the literature includes only scarce mentions at best. **Methods:** This is a descriptive microanatomical study including 25 cadaver brains. Specimens from donors with neurological, psychiatric disorders or alcohol abuse were excluded. Surgical loops were used for harvesting. High-precision tools were employed to dissect and measure the anatomical landmarks under a surgical microscope. Each measurement was performed in three consecutive attempts and outliers were rejected. RStudio was used for statistical analysis. Distribution was evaluated employing the Shapiro–Wilk test. Normally distributed values were presented as mean and standard deviation, and others as median and interquartile range. **Results:** The age of the donors was 61.72 (±10.08) years. The distance from the anterior aspect of the foramen of Monro to the anterior margin of the mamillary body was 16.83 (±1.04) mm, and to the posterior margin was 16.76 (±1.9) mm. The distance from the anterior mamillary body margin to the infundibulum was 6.39 (±1.9) mm, to the optic recess was 8.25 (±1.84) mm, and to the apex of the vertebral artery was 5.05 (±1.62) mm. The distance from the anterior commissure to the brain aqueduct was 22.46 (±2.29) mm, and to the infundibulum was 13.93 (±2.54) mm. The mamillary body diameter was 4.91 (±0.34) mm in the anteroposterior and 4.21 (±0.48) mm in the cranio-caudal plane. The intraventricular segment was protruding by 1.63 (±0.46) mm. The diameter of the hypothalamus on the anterior margin of mamillary bodies was 1.37 (±0.75) mm, of the Liliequist membrane was 0.19 (±0.07) mm and of the lamina terminalis was 0.35 (±0.32) mm. **Conclusion:** The presented microanatomical measurements and correlations are expected to contribute to the improvement of ETV safety.

## 1. Introduction

Hydrocephalus is characterized by an excessive accumulation of Cerebro-Spinal Fluid (CSF) in the ventricular system, leading to an increase in intracranial pressure. Endoscopic third ventriculostomy (ETV) is considered an effective treatment for hydrocephalus in adults and in selected pediatric cases [1,2]. Some surgeons even consider it the treatment of choice, as it presents lower complication rates than shunting [1], making it an appealing technique. However, the scientific community is yet to reach a consensus regarding the exact pathophysiological mechanisms leading to hydrocephalus, or provide a sound explanation, rather than theories, for the reason the communication between the intraventricular and subarachnoid spaces will lead to a resolution [3]. Moreover, the anatomical knowledge of the region of the third ventricle is limited to the description of the structures and their morphological variations, with scarce reference to morphometric characteristics. The aim of this study is to supplement this knowledge gap and assist in the prevention of complications during ETV.

## 2. Materials and Methods

**Figure 1:**
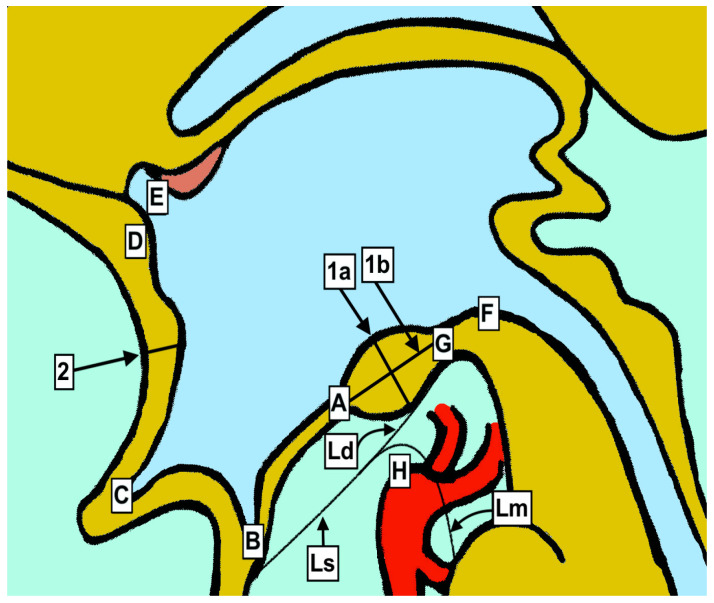
Graphical presentation of anatomical measurements. A—Third ventricle floor (hypothalamus) at anterior margin of mamillary bodies. B—Distal point of infundibular recess. C—Distal point of optic recess. D—Anterior commissure. E—Anterior border of the foramen of Monro. F—Entrance of the mesencephalic duct (Sylvius). G—Posterior mamillary body borderline. H—Basilar artery apex. Ls—Liliequist membrane—sellar. Ld—Liliequist membrane—diencephalic. Lm—Liliequist membrane—mesencephalic. 1a—Anteroposterior diameter between the most distal parts of mamillary bodies. 1b—Cranio-caudal diameter between the most distal points. 2—Cranio-caudal diameter of lamina terminalis.

**Figure 2:**
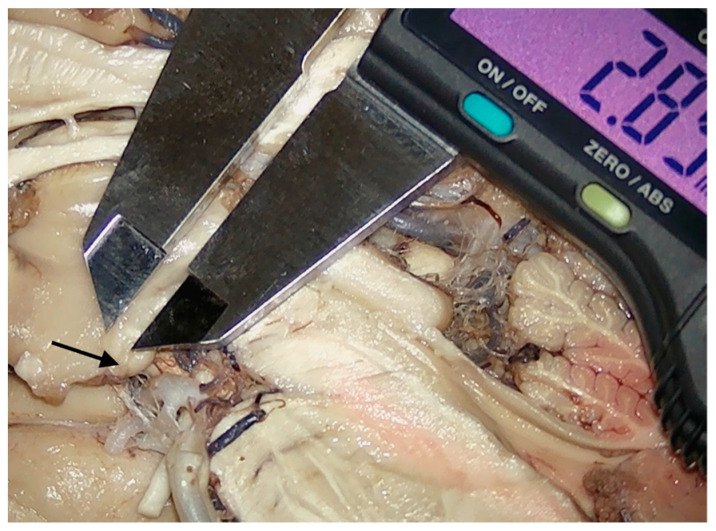
Presentation of the technique employed to provide a measurement of the intraventricular part of the mamillary body. The lower end of the instrument was placed on the level of the third ventricle floor, with the upper one being placed on the distal tip of the section projecting into the third ventricle. Arrow-head points toward the hypothalamus tissue limiting the intraventricular portion of the mamillary body.

This is a descriptive anatomical study including a total of 25 cadaveric brain specimens. All of the donors were of Caucasian race. None had a known neurological or psychiatric disorder, and their passing was not attributed to factors affecting the Central Nervous System. Cases with a clinical history or pathological evidence of alcohol abuse were also excluded, due to the potential effect on the size of the anatomical structures, particularly the mamillary bodies [4]. The harvesting was performed with the use of Zeiss EyeMag Medical Loops, with special attention to the areas being studied. The material was preserved by submersion in a 10% formalin solution for approximately 72 h before being examined and dissected under a Leica M530 OHX surgical microscope, using a microsurgical-instruments set. A high-accuracy Mitutoyo digital caliper was used for all anatomical measurements. In order to ensure correctness, each measurement was performed in three consecutive attempts and the outlier values were rejected. A Microsoft Office Excel data sheet was created including all the values (Table 1) of interest (Figure 1 and Table 2). The interventricular projection of the mamillary bodies was defined as the distance between the ventricular side of the hypothalamus and the most distal part of the mamillary body (Figure 2). Statistical analysis was conducted employing the RStudio 2022.12.0+353 software (Supplementary S1). Distribution was evaluated employing the Shapiro–Wilk test with a chosen alpha level of 0.05. Additional assessment was conducted through graphical presentation and kurtosis calculation. Normally distributed values were presented as mean and standard deviation (SD), while others were presented as median and interquartile range (IQR).

## 3. Results

The vast majority of cadaveric specimens included in the study were acquired from male donors (22/25 (88%)). This discrepancy was related to availability. The mean age of donors was 61.72 (±10.08). All segments of the Liliequist membrane including the sellar, diencephalic and mesencephalic parts were recognized in all of the specimens during the dissection. All were membranous and none presented any web-like pattern. They were translucent and in continuity with the pontomesencephalic and pontomedullary membranes. The oculomotor nerve was entirely covered on its course to the cavernous sinus. The hypothalamus in front of the mamillary bodies (the target place for the creation of stoma) was found to be translucent in 6 (24%) and solid in the rest, while lamina terminalis was found to be translucent in 16 (64%) of them. The interthalamic commissure was absent in nine (36%) specimens. All of the specimens presented a complete vascular circle of Willis, with no detectable anatomical alterations. A descriptive table including all parameters of interest is presented (Table 3.)

## 4. Discussion

**Figure 3:**
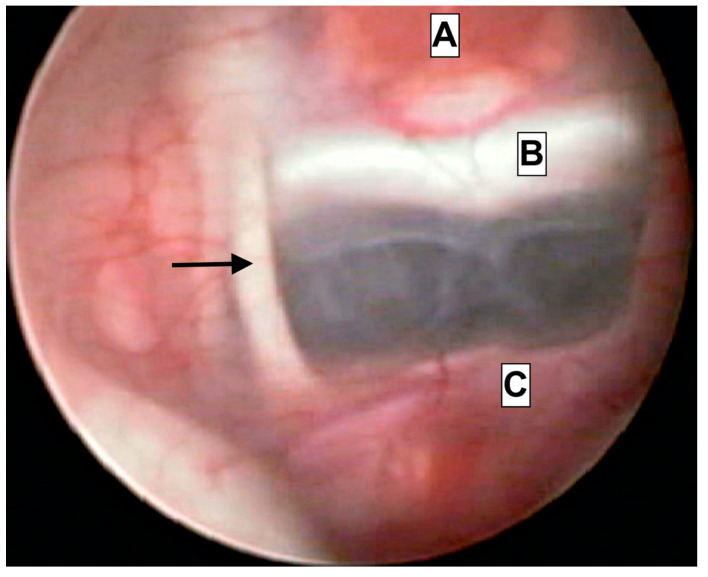
Endoscopical caption from intraoperative footage of a patient treated due to acute hydrocephalus. The close proximity of anatomical structures is presented. A—Hypophyseal tissue. B—clivus. C—Basilar artery apex. Arrowhead—Oculomotor nerve (III).

ETV is becoming the treatment of choice for adults and selected cases of children with hydrocephalus. Our awareness of the micro-anatomical measurements of the third ventricle region is limited. Detailed knowledge is mandatory in order to avoid intraoperative complications associated with the close proximity of important endocrine, vascular and neural structures. The reports of such events are scarce, but the results may be catastrophic, including hormonal abnormalities after pituitary gland trauma; neurologic disfunction due to thalamic or oculomotor trauma; and even death due to basilar, posterior communicating or posterior cerebral artery trauma [5,6]. Surgeons may, in some cases, be able to recognize the anatomy through an almost-transparent third ventricle floor (Figure 3); however, they will predominantly have to rely on their anatomical knowledge of the region.

**Figure 4:**
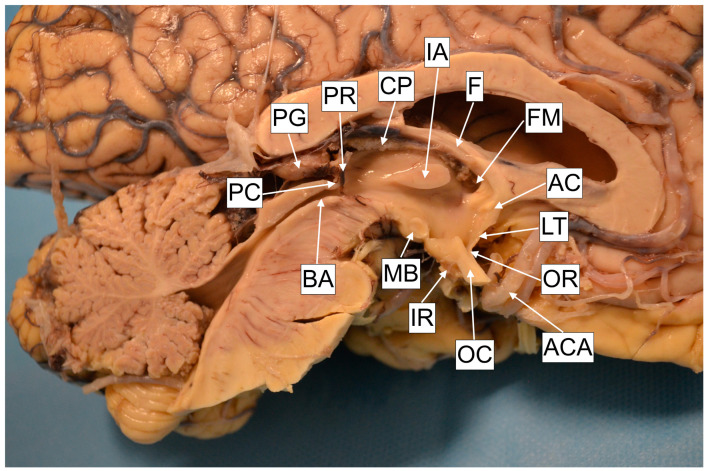
Cadaver specimen. AC—Anterior commissure. LT—Lamina terminalis. OR—Optic recess. ACA—Anterior cerebral artery. OC—Optic recess. IR—Infundibular recess. MB—Mamillary body. BA—Brain Aqueduct. PC—Posterior commissure. PG—Pineal gland. PR—Pineal recess. CP—Choroid plexus. IA—Interthalamic adhesion. F—Fornix. FM—Foramen of Monro.

**Figure 5:**
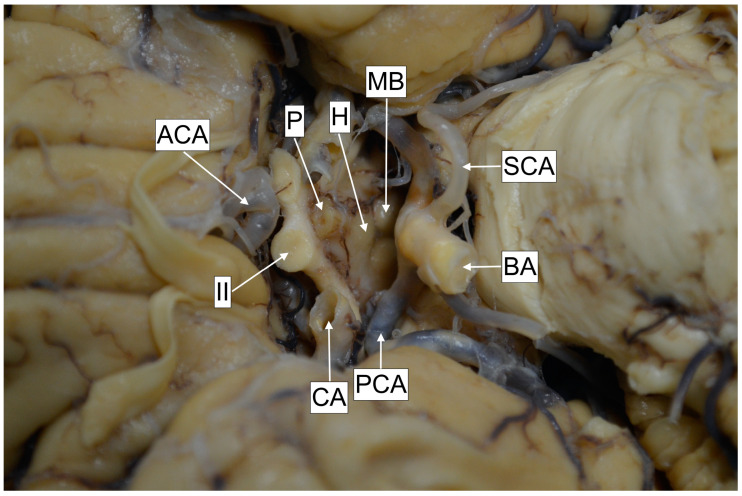
Cadaver specimen. MB—Mamillary bodies. SCA—Superior cerebellar artery. BA—Basilar artery. PCA—Posterior cerebral artery. CA—Carotid artery. II—Optic nerve. ACA—Anterior cerebral artery. P—Pituitary gland. H—Hypothalamus (segment between mamillary bodies and optic chiasm).

**Figure 6:**
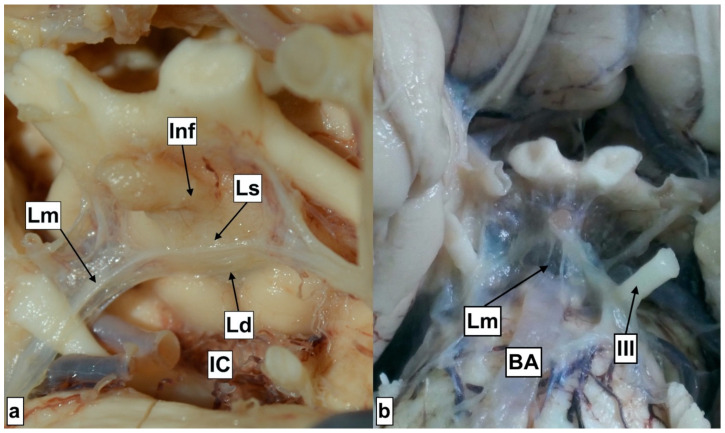
Liliequist membrane segments. IC—Interpeduncular cistern Ld—Diencephalic segment. Lm—Mesencephalic segment Ls—Sellar segment Inf—Infundibulum. III—Oculomotor nerve Image (**a**) with open Interpeduncular cistern. Image (**b**) with the Lm segment overlying the Interpeduncular cistern, covering the basilar artery.

The third ventricle presents a narrow and vertically oriented space connecting the lateral ventricles and the cerebral aqueduct. The rostral wall is formed out of the anterior commissure, in continuity with the lamina terminalis, which attaches to the anterior edge of the optic chiasm. The floor is formed by the hypothalamus, which extends from the mid-optic chiasm to the caudal part of the mamillary bodies. The lateral wall is formed inferiorly by the hypothalamus and superiorly by the thalamus. Thalami may present an interthalamic adhesion, bridging them at the superior–posterior part. The caudal wall is formed out of the posterior commissure and the pineal recess, and the roof is formed out of the tela choroidea, situated beneath the crura of the fornix (Figure 4). The distance from the anterior border of the foramen of Monro to the anterior aspect of the mamillary bodies was 16.83 (±1.04) mm, and this is nearly the distance a surgeon will have to advance the ventriculoscope after passing the foramen. The posterior aspect of the mamillary bodies is located 16.76 (±1.9) mm from the foramen of Monro, thus these two distances are almost equal, probably due to the obtuse angle of the third ventricle floor. Investigation of the thalami revealed that the interthalamic adhesion was absent in nine (36%) of the specimens. The literature points toward a relatively equal (30%) incidence in the general population [7]. The exact role of this anatomical formation has not yet been defined; however, it has been suggested that its presence may affect neurocognitive function, with some authors correlating its absence with a higher incidence of psychotic disorder development [8]. None of the donors had a history of psychiatric disorder. The mamillary bodies are considered a landmark for orientation. They are positioned on the posterior–inferior part of hypothalamus and present a pair of ellipsoids containing the lateral and medial brainstem nuclei. Their diameter is approx. 5 mm [9,10]. In agreement with the literature, we recognized their shape to be ellipsoid, thus we registered the two most distal diameter values on anteroposterior and cranio-caudal planes, yielding results similar to those presented in the literature, with the anteroposterior diameter measured at 4.91 (±0.34) mm and the cranio-caudal at 4.21 (±0.48) mm. The intraventricular part of the mamillary body was protruding for 1.63 (±0.46) mm. Anterior to the mamillary bodies lies the infundibular recess. This presents a styloid extension of the third ventricle, which may project as a canal through the entire pituitary gland. The distance from the mamillary body to the infundibular recess, thus from the pituitary stalk, was 6.39 (±1.9) mm. This particular region of the hypothalamus is the point of interest for the fenestration, which should be placed close to the anterior margin of the mamillary bodies. In case of distal placement, the pituitary gland tissue will be recognized beneath the stoma, instead of the clivus. However, we should furthermore highlight the close relationship of this point with the vascular structures of the posterior circulation (Figure 5). The distance from the point of fenestration to the apex of the basilar artery was found to be 5.05 (±1.62) mm. We have to keep in mind that this distance may be even smaller in patients with ventricular dilatation, and that the floor of the third ventricle may be in direct contact with the apex of the basilar artery and the posterior cerebral arteries (Figure 3). There has been a variety of anatomical variations described regarding the Willis loop both in children and adults [11,12]; however, none were recognized during our anatomical dissections. This could be associated with the study size; however, based on the assumption that more than 50% of the population is expected to express anatomical variations of the loop, we anticipated to identify at least one case. It is possible that the incidence of these variations is lower in our geographical region. The correlation of anatomical blood vessel variations with race and ethnicity have already been suggested [13]. However, robust multicenter anatomical and radiological studies should be conducted in order to draw a definitive conclusion. The diameter of the third ventricle floor, approximately on the target region for placing the stoma (Figure 1A), was 1.37 (±0.75) mm, and in 24% of the cases was translucent. Beneath the third ventricle lies the membrane of Liliequist. This partially trabecular and partially dense structure presents a projection of the arachnoid membrane [14]. It consists of three segments, which separate the chiasmatic, interpeduncular and prepontine cisterns [15]. All of the structures, including the sellar, diencephalic and mesencephalic segments, were recognized during the dissections (Figure 6).

They were all membranous and they converged to a single point, forming a thicker layer that can be also recognized intraoperatively (Supplementary S2). The sellar segment attaches to the superior edge of the dorsum sellae and to the mesencephalic and diencephalic segments, forming a junction. The sellar segment was resected during the harvesting procedure. The mesencephalic segment is additionally attached to the oculomotor nerve, to the midbrain and the pons, while the diencephalic further attaches to the oculomotor nerve and the hypothalamus. The oculomotor nerve seems to be completely surrounded by the membranes until the cavernous sinus. We could argue that the sellar segment presents a portion of one of the other two, as a definitive demarcation line was not detected at the junction level. The literature describes anatomical variations with the absence of one of the segments, or morphological variations (web-like consistency) [16]. The diameter of the diencephalic segment was found to be 0.19 (±0.0.07). The fenestration of this membrane is mandatory during the conduction of ETV. It is expected that ETV would result in the communication between the intraventricular and subarachnoid spaces; however, the fenestration of all layers could potentially create a communication with the subdural space as well. Lamina terminalis fenestration is described as a viable alternative to ETV [17]. Anatomical studies have assessed the height (≈8.25 mm), the base (≈12.81 mm) and the area (≈52.84 mm^2^) [18,19]; however, the diameter has not been appraised. This study presented that in 64% of the cases, the lamina was translucent, while the others were solid. The literature points toward a predominance of transparent (62.5%) when compared to translucent (31.3%) [19] regarding the optical consistency. The diameter of the lamina in our study is 0.35 (±0.32), and thus we detect that it presents a significant variation regarding its thickness. We would like to highlight that according to these results, lamina terminalis is the thinnest of the structures forming the walls of the third ventricle, something that could explain the spontaneous fenestration in rare cases of patients with high intracranial pressure. Other parameters providing significant information for the size of the third ventricle are the distance from the entrance of the cerebral (Sylvian) aqueduct to the anterior commissure, the distance from the infundibular recess to the anterior commissure, and the distance from the mamillary body to the optic recess. This could be employed in the diagnosis of hydrocephalus alongside other diagnostic tools, such as the widening of the third ventricle recess, the upward displacement of the corpus callosum, and the decreased mammillopontine distance [20]. It could also be employed for the detection of other conditions as well, for instance, as a prognostic factor for dementia, known to be associated with the dilatation of the third ventricle [21].

## 5. Conclusions

The presented microanatomical measurements and correlations are expected to contribute to the better understanding of the anatomical characteristics of the third ventricle floor, contributing to the improvement of safety during the conduction of ETV.

## Figures and Tables

**Table 1:** Measured anatomical structures and distances.

Anatomical Points of Interest		Correlation with Figure 1
Mamillary body anteroposterior diameter		1a
Mamillary body cranio-caudal diameter		1b
Intraventricular projection of mamillary body		1a (Figure 2)
Ventricle floor diameter—front mamillary border		A
Liliequist membrane diameter		Ld
Lamina terminalis diameter		2
Anterior border of mamillary body	Anterior border of foramen of Monro	A-E
Posterior border of mamillary body	Anterior border of foramen of Monro	G-E
Anterior border of mamillary body	Infundibular recess	A-B
Anterior border of mamillary body	Basilar artery apex	A-H
Anterior border of mamillary body	Optic recess	A-C
Infundibular recess	Anterior commissure	B-D
Sylvian aqueduct entrance	Anterior commissure	F-D

**Table 2:** Row-data of metrics (in mm) according to Figure 1 and Table 1.

*Cadavers*	*1a*	*1b*	*1a Figure 2*	*A*	*Ld*	*2*	*A-E*	*G-E*	*A-B*	*A-H*	*A-C*	*B-D*	*F-D*
*C1*	4.86	4.94	2.11	0.89	0.13	0.10	17.22	19.21	7.95	4.47	7.04	13.93	23.64
*C2*	5.26	3.93	1.29	1.73	0.16	0.25	18.43	17.29	5.23	2.50	4.78	15.47	21.34
*C3*	4.74	3.67	1.39	1.48	0.24	0.50	17.12	16.20	7.06	2.71	6.49	15.59	22.50
*C4*	4.97	4.10	0.95	1.56	0.14	0.33	16.25	14.97	2.47	2.68	6.42	14.40	17.52
*C5*	5.07	3.93	1.44	1.62	0.12	0.37	18.17	17.18	3.41	5.29	8.90	12.24	25.69
*C6*	4.90	4.45	1.20	1.43	0.08	0.34	16.37	14.40	5.19	4.62	4.97	12.89	20.40
*C7*	4.72	4.07	1.84	1.61	0.25	0.31	13.03	14.55	6.58	4.01	8.58	12.71	18.70
*C8*	5.07	4.19	1.55	1.72	0.31	0.27	16.69	17.23	6.94	3.30	9.29	15.50	25.44
*C9*	5.38	4.17	2.27	0.50	0.19	0.21	16.73	18.50	6.95	4.66	8.68	11.71	26.01
*C10*	5.22	4.37	0.91	1.70	0.11	0.29	12.56	12.08	3.62	3.1	5.04	14.78	23.43
*C11*	5.06	4.12	2.15	0.26	0.21	0.38	18.97	20.22	8.60	5.84	10.79	15.49	22.50
*C12*	4.38	4.03	1.47	0.50	0.14	0.14	13.34	13.74	10.83	6.66	10.36	11.72	20.90
*C13*	4.99	4.12	1.63	0.59	0.27	0.19	16.63	17.63	7.33	7.71	11.38	11.88	26.65
*C14*	4.52	3.60	1.84	0.34	0.31	0.26	16.57	16.72	9.53	6.45	10.86	14.69	23.30
*C15*	4.20	3.25	1.38	0.39	0.27	0.12	14.58	14.06	4.70	6.30	10.05	10.77	19.77
*C16*	5.69	4.90	2.10	1.77	0.09	1.16	16.83	17.02	5.60	6.62	9.63	14.72	24.70
*C17*	4.53	4.48	1.15	1.37	0.21	0.54	17.34	17.57	6.05	4.37	9.26	11.64	22.34
*C18*	5.02	4.97	1.98	0.86	0.15	0.35	16.85	17.98	5.14	6.73	8.73	12.07	19.87
*C19*	4.49	4.53	1.58	1.11	0.19	0.95	17.15	16.97	6.11	3.83	8.48	13.16	22.01
*C20*	4.62	4.06	2.01	0.94	0.12	0.87	16.83	17.24	4.97	5.58	7.93	14.65	21.89
*C21*	5.01	4.88	1.65	1.59	0.22	1.03	17.79	17.29	6.07	2.94	6.29	15.21	23.12
*C22*	5.29	5.04	1.44	1.37	0.17	0.65	17.59	17.71	7.44	6.89	8.48	13.35	20.98
*C23*	4.91	3.47	1.24	1.26	0.19	0.58	17.42	17.07	6.47	5.04	8.18	14.72	21.69
*C24*	4.74	4.04	1.27	1.03	0.21	0.73	16.38	16.54	7.08	6.94	7.39	13.89	24.43
*C25*	5.09	3.98	2.89	1.68	0.24	0.43	18.24	19.67	8.31	7.07	8.17	15.62	22.77

**Table 3:** Descriptive results table (All parameters of interest are presented employing the * mean/SD and ^‡^ median/IQR based on distribution. All variables are expressed in mm, except for the age being presented in years).

Parameters	Shapiro (*p*)	Descriptives		Min	Max
**Age**	0.1012	61.72 *	(±10.08)	34	75
**1a**	0.9704	4.91 *	(±0.34)	4.2	5.69
**1b**	0.2415	4.21 *	(±0.48)	3.2	5.04
**1a Figure 2**	0.2881	1.63 *	(±0.46)	0.91	2.89
**A**	0.01241	1.37 ^‡^	(±0.75)	0.26	1.77
**Ld**	0.6264	0.19 *	(±0.07)	0.08	0.31
**2**	0.01157	0.35 ^‡^	(±0.32)	0.10	1.16
**A-E**	0.001672	16.83 ^‡^	(±1.04)	12.56	18.97
**G-E**	0.1607	16.76 *	(±1.9)	12.08	20.22
**A-B**	0.9854	6.39 *	(±1.9)	2.47	10.83
**A-H**	0.1148	5.05 *	(±1.62)	2.50	7.71
**A-C**	0.4471	8.25 *	(±1.84)	4.78	11.38
**B-D**	0.03542	13.93 ^‡^	(±2.54)	10.77	15.62
**F-D**	0.9865	22.46 *	(±2.29)	17.52	26.65

## Data Availability

The complete dataset is available in the manuscript and the statistical analysis is represented in the Supplementary Materials.

## Supplementary Materials

The following supporting information can be downloaded at: https://www.mdpi.com/article/10.3390/brainsci13040580/s1, Supplementary S1: R-studio statistical program lines and database, Supplementary S2: Video presentation of Third Ventriculostomy.

## Statement

This study is part of the doctoral dissertation Micro-anatomical, imaging and neuroendoscopic correlations of the 3rd ventricle floor conducted by the leading author at the Aristotle University of Thessaloniki, School of Medicine.
